# UTAUT for HSS: initial framework to study health IT adoption in the developing countries

**DOI:** 10.12688/f1000research.13798.1

**Published:** 2018-01-23

**Authors:** Anis Fuad, Chien-Yeh Hsu

**Affiliations:** 1 Department of Biostatistics, Epidemiology and Population Health , Faculty of Medicine, Public Health and Nursing, Universitas Gadjah Mada, Yogyakarta, Indonesia; 2 Center for Health Policy and Management, Faculty of Medicine, Public Health, and Nursing, Universitas Gadjah Mada, Yogyakarta, Indonesia; 3 Department of Information Management, National Taipei University of Nursing and Health Sciences, Taipei, Taiwan; 4 College of Public Health, Taipei Medical University, Taipei, Taiwan

**Keywords:** UTAUT, health system strengthening, developing countries

## Abstract

Unified Theory of Acceptance and Use of Technology (UTAUT) is an integrative concept that has been used widely to measure IT adoption. However, a recent study in a developing country concluded that UTAUT is not adequate in predicting IT adoption within the context of health system strengthening (HSS). It has been suggested that context-specific dimensions to modify UTAUT should be considered. The objective of this paper is to propose an extension of the theory, called UTAUT for HSS, as a reference for contextualizing health system variables for health IT adoption studies in the developing countries. We combined the multi-level framework of UTAUT with WHO health system building blocks. Modification of the original multi-level framework was performed on the 3 levels. i.e:  the higher-level contextual factors, middle-level, and individual-level contextual factors. Based on this, we propose a modified multi-level framework of technology acceptance and use for health system strengthening setting (UTAUT for HSS).  Given the complexities of health systems, more thoughts regarding the methodologies will be useful to enrich this initial framework.  Commentaries and discussions are invited for improvement, before implementation to obtain more complete story of health IT adoption in the low resources setting.

## Introduction

Improved health status of the population can only be achieved by a strengthened health system. Health system strengthening (HSS) efforts require a sound and reliable health information system ^1^. Consequently, adoption of information technology (IT) becomes inevitable to improve data use, program management, and health services. Despite the high expectation, adopting IT in a health system is far from simple. Similar interventions can produce opposite results in different health system environments. From non-adoption and abandonment on one side to a well adopted and sustainable use in another side ^2^. Multiple factors, individual to social, are involved and interact each other influencing the behavior to adopt the technologies.

Unified Theory of Acceptance and Use of Technology (UTAUT) is an integrative concept that has been used widely to measure IT adoption since the original paper introduced in 2003 ^3^. UTAUT formulated four core determinants (performance expectation, effort expectation, social influence, and facilitating conditions) and up to four moderators (gender, age, experience, and voluntariness of use) influencing individual behavior to adopt and use IT. Scopus scholarly database recorded 8939 citations to UTAUT as of 16 January 2018. Although this has been cited in many fields for more than a decade, nevertheless, only a limited number of papers contributed to integration of UTAUT with external theories or extend the original conceptual framework ^4^.

Until 16 January 2018, in PubMed, about 106 articles specifically mentioned UTAUT. Those studies were conducted in developed and developing countries. A recent study from Cameroon where public hospitals clinicians adopted hospital information systems, provided an interesting insight. The authors concluded that UTAUT is not adequate in predicting health IT adoption in a developing country setting. For researchers aiming to measure health IT adoption in developing countries, they suggested considering context-specific dimensions in modifying UTAUT ^5^.

This paper aims to contextualize health system dimensions to study IT adoption using UTAUT framework in the low-resources setting. We try to integrate UTAUT with health system frameworks of WHO ^1^ with an expectation to invite more debates regarding the appropriate models to evaluate health IT intervention for health system strengthening initiatives, particularly in the developing countries.

## UTAUT for HSS: extension of UTAUT for health system strengthening

Cognizant of the low number of UTAUT theoretical integration or extension research, UTAUT authors structured four promising extensions of UTAUT. These include new exogenous mechanisms, new endogenous mechanisms, new moderation mechanisms, and new outcome mechanisms. New exogenous mechanisms represent the impacts of external predictors to the four core determinants (i.e., performance expectation, effort expectation, social influence, and facilitating conditions). New endogenous mechanisms could include: 1) new predictors’ impact on the two endogenous variables (i.e., behavioral intention and use behavior) or 2) the enhancement of the four core determinants and the two endogenous variables. New moderating mechanisms involve new moderating effects complemented to the original UTAUT. New outcome mechanisms refer to the new effect of behavioral intention and technology use. All the four extensions are integrated into a multi-level framework consisting of higher-level contextual factors, a middle level containing the baseline model of UTAUT, and individual level contextual factors ^4^.

This new framework offers opportunities to contextualize system-wide variables within the health system building blocks as predictors influencing the status of health IT adoption. Characteristics of developing countries (in national, sub-national or service delivery level) are very relevant to be embedded in the framework. A recent study identified four contextual factors in developing countries, namely hierarchical roles, aid funding, corruption, and competing priorities that potentially influence the success of health information strengthening ^6^. Developing countries also face a shortage of health workers in rural areas, the variable quality of care, lack of patient compliance, and fraud ^7^.

The above issues can be classified according to the six health building blocks i.e.1) service delivery, 2) health workforce, 3) information, 4) medical products, vaccines & technologies (MPVT), 5) financing, and 6) leadership and governance ^1^. Progress and characteristics of the information building block in a country depend on the following components: 1) leadership and governance, 2) strategy and investment, 3) services and applications, 4) standards and interoperability, 5) infrastructure and 6) workforce ^8^.

**Figure 1.**
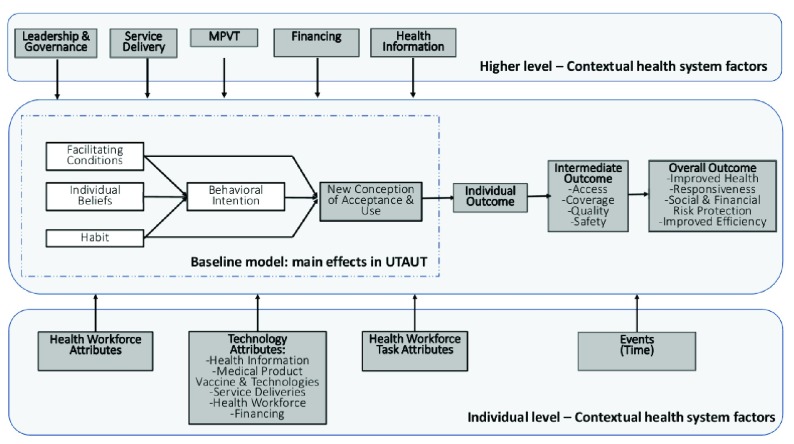
UTAUT for HSS: a modified multi-level framework of technology acceptance and use for health system strengthening context.

Therefore, we propose UTAUT for HSS, a modified multi-level framework of technology acceptance and use for health system strengthening setting ( Figure 1). We combined the UTAUT multi-level framework ^4^ with WHO health systems building block ^1^ to address the issue of contextualizing health system variables to improve health IT adoption models. Compared to the original framework ^4^ we performed a modification at the three levels. In the higher-level, we have changed the original attributes (environment, organization, and location) with five health system building blocks. In the middle-level, we have changed the original new outcome phenomenon with three related outcomes namely individual (health workforce) outcome, intermediate (health system) outcome and overall (health system) outcome. In the individual-level, we specifically mention health workforce and health IT attributes. E-health building blocks as proposed by WHO-ITU ^8^ could be embedded into the existing health system building blocks.

Another health IT research in a developing country reported process, result, and policy implication of electronic medical record (EMR) readiness assessment on 381 health facilities in Kenya ^9^. This report is an example of event-time related activities before the implementation of health IT intervention. Since IT adoption is a time-dependent process, this current framework allows for multiple assessments periodically that could be compared to measure the progress of adoption.

Although promising, researchers wanting to use UTAUT in HSS setting should consider the potential limitations. Many UTAUT studies depend on a restricted user type, such as the office workers. In health system context, involving a different type of user including patients, various type of health workers and policymakers will enrich the big picture. UTAUT has long been recognized as a functionalist-institutionalist theory that focuses on systemic stability. Combining with other interpretive theories will be useful to better understand the complex social and symbolic reality of health IT adoption ^10^. This will unsurprisingly trigger the discussion of the pro and con of using other methods such as Implementation Research, Mixed Methods, and Qualitative Studies to apply UTAUT within the milieu of HSS setting.
