# Supplementary material for: UTAUT for HSS: initial framework to study health IT adoption in the developing countries
Source: F1000Res. 2018 Jan 23;7:101. [Version 1] doi: 10.12688/f1000research.13798.1 (PMC6069728; doi:10.12688/f1000research.13798.1)
Supplement: Peer review history

# Peer review: Referee response for version 1

**DOI:** 10.5256/f1000research.15000.r36284

**Published:** 2018-07-30

**Authors:** Eric D. Perakslis

**Affiliations:** 1 Department of Biomedical Informatics, Harvard Medical School, Boston, MA, USA

The article would benefit from the inclusion of definition and context for health information technology.  For example, by HIT, does the author describe full EMR implementations, which are quite complex, versus other HIT tools?  Is the proposed framework suitable and needed to all forms of HIT efforts?

 Second, the author briefly describes other methodologies at the end of the paper.  It would be helpful to consider a few other methods in a bit more detail to compare and contrast the potential of the proposed/modified UTAUT approach.

 Lastly, the piece could benefit from examples of the proposed recommendations, even if hypothetical, that would provide some practical grounding and understanding of the proposed enhancements to the UTAUT framework.

I have read this submission. I believe that I have an appropriate level of expertise to confirm that it is of an acceptable scientific standard.

# Peer review: Referee response for version 1

**DOI:** 10.5256/f1000research.15000.r36269

**Published:** 2018-07-27

**Authors:** Vijayan Kumara Pillai

**Affiliations:** 1 University of Texas at Arlington, Arlington, TX, USA

This article makes valuable contribution to the theory of UTAUT. Even though the extension suggested is by no means surprising, the authors have explained it well and conveyed their ideas both diagrammatically and in words. Only criticism is that, they have not reported any empirical support for the theoretical extension proposed.

I have read this submission. I believe that I have an appropriate level of expertise to confirm that it is of an acceptable scientific standard.
